# Commentary: Preliminary evaluation of an analog procedure to assess acceptability of intimate partner violence against women: the Partner Violence Acceptability Movie Task

**DOI:** 10.3389/fpsyg.2017.01766

**Published:** 2017-10-09

**Authors:** Antonio Iudici, Elena Faccio, Gianluca Castelnuovo

**Affiliations:** ^1^Department of Philosophy, Sociology, Education and Applied Psychology, University of Padova, Padua, Italy; ^2^Department of Psychology, Catholic University of Milan, Milan, Italy; ^3^Psychology Research Laboratory, Istituto Auxologico Italiano IRCCS, Ospedale San Giuseppe, Verbania, Italy

**Keywords:** intimate partner violence, violence, movie task, evaluation, acceptability of violence, women with disability, health

The acceptability of intimate violence as it regards women is a problem which researchers and institutions studying global health have questioned for some time now. Research has focused on this aspect as a determining factor in the perpetration of violent actions. The process involves both the abusers (Bryant and Spencer, 2003; Taylor and Sorenson, 2005; Waltermaurer, 2012), the entire community (Gracia and Herrero, 2006; Frye, 2007), and the victims directly (Flood and Pease, 2009; Kogut, 2011; Rizo and Macy, 2011; Eckhardt et al., 2012).

It has been observed that the latter can come to think that intimate violence is normal, that it is accepted if one is in certain conditions, such as having a very low income (Smith, 2008), being unemployed (Mitra and Mouradian, 2014) or having a disability (Iudici, 2015; Iudici and Renzi, 2015). Detecting the acceptability of violence, however, is a rather difficult task. Yet this is exactly why it deserves even more attention, with the aim of reducing the wide range of health implications which IPV brings, such as the risk of homicide (Stöckl et al., 2013), gastrointestinal disorders, hypertension, a weakened immune system, symptoms of depression, self-destructive thoughts, anxiety, insomnia, feelings of guilt and isolation (Campbell, 2002; Fanslow and Robinson, 2004; Ellsberg et al., 2008; Garcia-Moreno and Watts, 2011; European Union Agency for Fundamental European Union Agency for Fundamental Rights, 2014; Hasan et al., 2014).

The authors of the work “Preliminary evaluation of an analog procedure to assess acceptability of intimate partner violence against women: the Partner Violence Acceptability Movie Task” (Gracia et al., 2015) deserve credit for investigating the acceptability of the violence through PVAM, a work programme to recognize partner violence. This analogic procedure is based on responses to video clips which represent physical aggression against women. The time delay in reacting to the video clip indicates the justification level of the violence, thus a greater degree of acceptability. Regardless of how much this method could be improved, its value lies in being the first tool-based attempt to intercept the acceptability of violence, which is one of the factors that leads its actualisation.

In addition, the specific benefit of the tool is that of detecting the implicit aspects (Wilson et al., 2000; Fazio and Olson, 2003; Eckhardt et al., 2012) of acceptability, that is, aspects which are hard to gather through interviews and other tools. Also, considering that reaction times are less dependent on language, PVAM can make it possible to go beyond other limits found in other tools, such as the IAT (Holtzworth-Munroe and Stuart, 1994; Messner and Vosgerau, 2010). This could allow clinics and proposed services to integrate the data which they collect through other tools, such as interviews.

The results obtained from the use of the tool by Gracia et al. (2015) offer an additional, important, key piece of data: they have detected the justifying processes used by the abusers who carry out the violence, as was even noted by the scientific research (European Commission, 2010; Entilli and Cipolletta, 2017). This tool is also important as it can be a first step in scientific research to go beyond a few limits and distortions deriving from the current methods of data collection, which are almost always self-compiled (Eckhardt et al., 2012).

The analogic component of the tool can thus become a new channel in the interception of a problem which we know to be mostly hidden (Curry et al., 2011; Iudici et al., 2017). If such a requirement is necessary for women, it is even more necessary for women with disabilities, who are twice as vulnerable to intimate partner violence (Gammino et al., 2016).

Scientific evidence shows that disabled women are more likely to come up against different types of domestic violence, in a greater number compared to other women (Nosek et al., 2006; Barrett et al., 2009; Healey et al., 2013), for longer periods of time, duration and severity (Saxton et al., 2001; Brownridge, 2006; Breiding and Armour, 2015). The situation is particularly grave in that the abusers often use ways to justify their behavior (Saxton et al., 2001), stereotypes about disabled women (Ballan and Freyer, 2012; Faccio et al., 2014), psychological blackmail to bring about the acceptance of the harassment and violence (Plummer and Findley, 2012) and in implicit form (Eckhardt and Crane, 2014). In parallel, many disabled women believe it's normal to be treated violently, and for this reason they justify the behavior of their partner (Marra, 2009). Indeed, the risk of social exclusion which many disabled women face also brings with it a difficulty in recognizing if that which they are subjected to is considered violence (Holtzworth-Munroe et al., 2010). The risk of losing a few basic personal assistance services (sanitation/hygiene services, getting dressed and other intimate activities) can bring about a greater level of acceptance of abuse (Smith, 2008; Castelnuovo, 2010).

As a result, tools such as PVAM and their potential developments can be a determining factor in early detection of those who may be exposed to IPV. And it is only through the development of similar and new tools which we can encourage the effective promotion of the health of disabled women and an increase in their protection (Castelnuovo et al., 2003), especially considering the vulnerability a category which, precisely due to the need for stable family support, is increasingly exposed to intimate violence.

## Author contributions

AI has dealt with the Conception or design of the work. Data collection, analysis and interpretation, Drafting the article and the final Critical revision of the article have been elaborated by AI, EF, and GC.

### Conflict of interest statement

The authors declare that the research was conducted in the absence of any commercial or financial relationships that could be construed as a potential conflict of interest.

## References

[B1] Ballan M. S. Freyer M. B. (2012). Self-defence among women with disabilities: an unexplored domain in domestic violence cases. Violence Against Women 18, 1083–1107. 10.1177/1077801212461430 22996630

[B2] Barrett K. A. O'Day B. Roche A. Carlson B. L. (2009). Intimate partner violence, health status, and health care access among women with disabilities. Women Health Iss. 19, 94–100. 10.1016/j.whi.2008.10.005 19272559

[B3] Breiding M. J. Armour B. S. (2015). The association between disability and intimate partner violence in the United States. Ann. Epidemiol. 25, 455–457. 10.1016/j.annepidem.2015.03.017 25976023 PMC4692458

[B4] Brownridge D. (2006). Partner violence against women with disabilities: prevalence, risk, and explanations. Violence Against Women 12, 805–822. 10.1177/1077801206292681 16905674

[B5] Bryant S. A. Spencer G. A. (2003). University students' attitudes about attributing blame in domestic violence. J. Fam. Violence 18, 369–376. 10.1023/A:1026205817132

[B6] Campbell J. C. (2002). Health consequences of intimate partner violence. Lancet 359, 1331–1336. 10.1016/S0140-6736(02)08336-8 11965295

[B7] Castelnuovo G. (2010). No medicine without psychology: the key role of psychological contribution in clinical settings. Front. Psychol. 1:4. 10.3389/fpsyg.2010.00004 21833187 PMC3153736

[B8] Castelnuovo G. Gaggioli A. Mantovani F. Riva G. (2003). New and old tools in psychotherapy: the use of technology for the integration of the traditional clinical treatments. Psychotherapy 40:33 10.1037/0033-3204.40.1-2.33

[B9] Curry M. A. Renker P. Robinson-Whelen S. Hughes R. B. Swank P. Oschwald M.. (2011). Facilitators and barriers to disclosing abuse among women with disabilities. Violence Victims 26, 430–444. 10.1891/0886-6708.26.4.430 21882667

[B10] Eckhardt C. I. Crane C. A. (2014). Male perpetrators of intimate partner violence and implicit attitudes toward violence: Associations with treatment outcomes. Cogn. Ther. Res. 38, 291–301. 10.1007/s10608-013-9593-5 25598562 PMC4294270

[B11] Eckhardt C. I. Samper R. Suhr L. Holtzworth-Munroe A. (2012). Implicit attitudes toward violence among male perpetrators of intimate partner violence: a preliminary investigation. J. Interpers. Violence 27, 471–491. 10.1177/0886260511421677 22333320

[B12] Ellsberg M. Jansen H. A. Heise L. Watts C. H. Garcia-Moreno C. (2008). Intimate partner violence and women's physical and mental health in the WHO multi-country study on women's health and domestic violence: an observational study. Lancet 371, 1165–1172. 10.1016/S0140-6736(08)60522-X 18395577

[B13] Entilli L. Cipolletta S. (2017). When the woman gets violent: the construction of domestic abuse experience from heterosexual men's perspective. J. Clin. Nurs 26, 2328–2341. 10.1111/jocn.13500 27505865

[B14] European Commission (2010). Domestic Violence against Women Report. Brussels: DG Justice.

[B15] European Union Agency for Fundamental Rights (2014). Violence against Women: An EU-Wide Survey. Luxembourg: Publications Office of the European Union.

[B16] Faccio E. Casini C. Cipolletta S. (2014). Forbidden games: the construction of sexuality and sexual pleasure by BDSM ‘players’. Cult. Health Sex. 16, 752–764. 10.1080/13691058.2014.909531 24828811

[B17] Fanslow J. Robinson E. (2004). Violence against women in New Zealand: prevalence and health consequences. N.Z. Med. J. 117:U1173. Available online at: http://www.nzma.org.nz/journal/117-1206/1173/15570342

[B18] Fazio R. H. Olson M. A. (2003). Implicit measures in social cognition research: their meaning and use. Annu. Rev. Psychol. 54, 297–327. 10.1146/annurev.psych.54.101601.145225 12172003

[B19] Flood M. Pease B. (2009). Factors influencing attitudes to violence against women. Trauma Violence Abuse 10, 125–142. 10.1177/1524838009334131 19383630

[B20] Frye V. (2007). The informal social control of intimate partner violence against women: exploring personal attitudes and perceived neighborhood social cohesion. J. Comm. Psychol. 35, 1001–1018. 10.1002/jcop.20209

[B21] Gammino G. R. Faccio E. Cipolletta S. (2016). Sexual assistance in Italy: an explorative study on the opinions of people with disabilities and would-be assistants. Sex. Disab. 34, 157–170. 10.1007/s11195-016-9435-y

[B22] Garcia-Moreno C. Watts C. (2011). Violence against women: an urgent public health priority. Bull. World Health Organ. 89, 2–2. 10.2471/BLT.10.085217 21346880 PMC3040024

[B23] Gracia E. Herrero J. (2006). Public attitudes toward reporting partner violence against women and reporting behavior. J. Marriage Fam. 68, 759–768. 10.1111/j.1741-3737.2006.00288.x

[B24] Gracia E. Rodriguez C. M. Lila M. (2015). Preliminary evaluation of an analog procedure to assess acceptability of intimate partner violence against women: the Partner Violence Acceptability Movie Task. Front. Psychol. 6:1567. 10.3389/fpsyg.2015.01567 26528220 PMC4600898

[B25] Hasan T. Muhaddes T. Camellia S. Selim N. Rashid S. F. (2014). Prevalence and experiences of intimate partner violence against women with disabilities in Bangladesh: results of an explanatory sequential mixed-method study. J. Interpers. Violence, 29, 3105–3126. 10.1177/0886260514534525 24860077

[B26] Healey L. Humphreys C. Howe K. (2013). Inclusive domestic violence standards: strategies to improve interventions for women with disabilities? Violence Victims 28, 50–68. 10.1891/0886-6708.28.1.50 23520832

[B27] Holtzworth-Munroe A. Stuart G. L. (1994). Typologies of male batterers: three subtypes and the differences among them. Psychol. Bull. 116:476. 7809309 10.1037/0033-2909.116.3.476

[B28] Holtzworth-Munroe A. Beck C. J. Applegate A. G. (2010). The mediator's assessment of safety issues and concerns (MASIC): a screening interview for intimate partner violence and abuse available in the public domain. Family Court Rev. 48, 646–662. 10.1111/j.1744-1617.2010.001339.x

[B29] Iudici A. (2015). Sexual harassment against people with mental disabilities in transit environments: implications for services and clinics, in Safety and Security in Transit Environments (London: Palgrave Macmillan), 328–343.

[B30] Iudici A. Renzi C. (2015). The configuration of job placement for people with disabilities in the current economic contingencies in Italy: social and clinical implications for health. Disabil. Health J. 8, 586–593. 10.1016/j.dhjo.2015.06.004 26233649

[B31] Iudici A. Bertoli L. Faccio E. (2017). The ‘invisible’ needs of women with disabilities in transportation systems. Crime Prevent. Commun. Saf. 19, 264–275. 10.1057/s41300-017-0031-6

[B32] Kogut T. (2011). Someone to blame: when identifying a victim decreases helping. J. Exp. Soc. Psychol. 47, 748–755. 10.1016/j.jesp.2011.02.011

[B33] Marra A. (2009). Ripensare alla disabilità tramite i disability studies in Inghilterra. Intersticios Revista de Pensamiento Critico 3, 79–99.

[B34] Messner C. Vosgerau J. (2010). Cognitive inertia and the implicit association test. J. Mark. Res. 47, 374–386. 10.1509/jmkr.47.2.374

[B35] Mitra M. Mouradian V. E. (2014). Intimate partner Violence in the relationship of men with disabilities in the United States: relative prevalence and Health correlates. J. Interpers. Violence 29, 3150–3166. 10.1177/0886260514534526 24860076

[B36] Nosek M. A. Hughes R. B. Taylor H. B. Taylor P. (2006). Disability, psychosocial, and demographic characteristics of abused women with physical disabilities. Violence Against Women 12, 838–850. 10.1177/1077801206292671 16905676

[B37] Plummer S. B. Findley P. A. (2012). Women with disabilities' experience with physical and sexual abuse: review of the literature and implications for the field. Trauma Violence Abuse 13, 15–29. 10.1177/1524838011426014 22070987

[B38] Rizo C. F. Macy R. J. (2011). Help seeking and barriers of Hispanic partner violence survivors: a systematic review of the literature. Aggress. Violent Behav. 16, 250–264. 10.1016/j.avb.2011.03.004

[B39] Saxton M. Curry M. A. Powers L. E. Maley S. Eckels K. Gross J. (2001). “Bring my scooter so i can leave you” a study of disabled women handling abuse by personal assistance providers. Violence Against Women 7, 393–417. 10.1177/10778010122182523

[B40] Smith D. (2008). Disability, gender and intimate partner violence: relationships from the behavioral risk factor surveillance system. Sex. Disabil. 26, 15–28. 10.1007/s11195-007-9064-6

[B41] Stöckl H. Devries K. Rotstein A. Abrahams N. Campbell J. Watts C.. (2013). The global prevalence of intimate partner homicide: a systematic review. Lancet 382, 859–865. 10.1016/S0140-6736(13)61030-2 23791474

[B42] Taylor C. A. Sorenson S. B. (2005). Community-based norms about intimate partner violence: putting attributions of fault and responsibility into context. Sex Roles 53, 573–589. 10.1007/s11199-006-9155-3

[B43] Waltermaurer E. (2012). Public justification of intimate partner violence: a review of the literature. Trauma Viol. Abuse 13, 167–175. 10.1177/1524838012447699 22643069

[B44] Wilson T. D. Lindsey S. Schooler T. Y. (2000). A model of dual attitudes. Psychol. Rev. 107, 101–126. 10.1037/0033-295X.107.1.101 10687404

